# PythiaStudio: a one-stop protein engineering platform powered by Pythia model suite

**DOI:** 10.1093/nar/gkag408

**Published:** 2026-05-15

**Authors:** Jinyuan Sun, Kelun Shi, Han Li, Yinglu Cui, Luoyi Wang, Bian Wu

**Affiliations:** State Key Laboratory of Microbial Diversity and Innovative Utilization, Institute of Microbiology, Chinese Academy of Sciences, Beijing 100101, China; College of Life Sciences, University of Chinese Academy of Sciences, Beijing 100049, China; State Key Laboratory of Microbial Diversity and Innovative Utilization, Institute of Microbiology, Chinese Academy of Sciences, Beijing 100101, China; College of Life Sciences, University of Chinese Academy of Sciences, Beijing 100049, China; Independent Researcher; State Key Laboratory of Microbial Diversity and Innovative Utilization, Institute of Microbiology, Chinese Academy of Sciences, Beijing 100101, China; Beijing Key Laboratory of Genetic Element Biosourcing & Intelligent Design for Biomanufacturing, Institute of Microbiology, Chinese Academy of Sciences, Beijing 100101, China; State Key Laboratory of Microbial Diversity and Innovative Utilization, Institute of Microbiology, Chinese Academy of Sciences, Beijing 100101, China; Beijing Key Laboratory of Genetic Element Biosourcing & Intelligent Design for Biomanufacturing, Institute of Microbiology, Chinese Academy of Sciences, Beijing 100101, China; State Key Laboratory of Microbial Diversity and Innovative Utilization, Institute of Microbiology, Chinese Academy of Sciences, Beijing 100101, China; College of Life Sciences, University of Chinese Academy of Sciences, Beijing 100049, China; State Key Laboratory of Green Biomanufacturing, Beijing University of Chemical Technology, Beijing 100029, China

## Abstract

Predicting how mutations affect protein stability and protein-protein binding affinity is crucial for protein engineering and drug development. Although several computational tools have been developed for these tasks, they often require specialized expertise and are difficult to integrate into unified workflows. Here, we present PythiaStudio (https://pythiastudio.wulab.xyz), a comprehensive web platform that integrates our recently developed Pythia, Pythia-PPI, and Pythia-Pocket models with complementary protein analysis tools. The platform enables users to predict mutational effects on protein stability and protein-protein binding affinity, ligand binding pocket, through an intuitive interface. Additional features include fitness and structure prediction. PythiaStudio provides interactive visualization tools, including mutation heatmaps, sortable result tables, and structure viewers. Importantly, the platform offers an integrated engineering workflow that combines stability and fitness predictions to guide rational protein design. We demonstrate the utility of this workflow through multiple cases, including different glycoside hydrolases and amidases. In these cases, the two-step computational redesign strategy successfully improved both thermostability and catalytic activity. PythiaStudio democratizes access to state-of-the-art deep learning-based protein engineering methods, enabling researchers without computational expertise to perform sophisticated protein engineering.

## Graphical Abstract

**Graphical Abstract:**
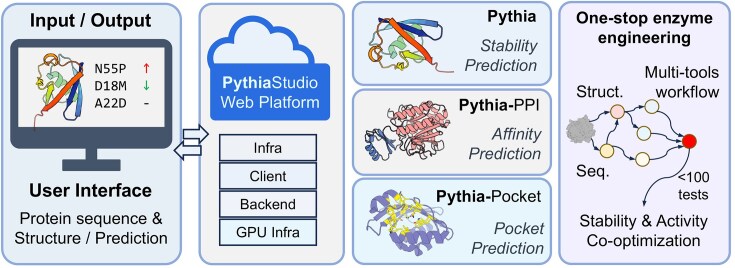

## Introduction

Proteins are fundamental molecular machines that execute diverse biochemical functions across all living organisms. Understanding how mutations affect protein properties is essential for fields ranging from disease mechanism elucidation to therapeutic protein development and industrial enzyme engineering [1–3]. Several web-based platforms have emerged to tackle specific aspects of protein engineering and selection. For example, the FireProt server automates thermostability design by identifying combinations of mutations that yield robust multiple-point mutants, integrating structural and evolutionary analyses in its workflow [4]. MutationExplorer provides an interactive environment for mapping sequence variants onto 3D structures with Rosetta-calculated energy changes highlighted per residue [5]. DeepMolecules focuses on enzyme–small molecule interactions, using ensemble models to predict likely substrates and even kinetic parameters (*K*_M_, *k*_cat_) for enzymes and transporters [6]. These web tools underscore the growing demand for reliable *in silico* prediction systems in protein engineering, but address largely non-overlapping aspects of the protein engineering process. MutationExplorer assists expert structural analysis of individual mutations but does not provide automated high-throughput screening, while DeepMolecules focuses on enzyme–substrate specificity rather than protein stability or binding affinity. No existing platform unifies mutation-induced stability prediction, protein–protein interaction analysis, active-site annotation, and evolutionary fitness scoring within a single integrated workflow.

Here, we introduce PythiaStudio, an integrated and high-throughput web platform that concurrently evaluates mutation impacts on protein stability, protein–protein binding affinity, and active-site features using advanced machine-learning models. Two key parameters that determine protein function are thermodynamic stability, quantified by the folding free energy ΔG_folding_), and binding affinity to interaction partners, described by the binding free energy ΔG_binding_). Single-point mutations can substantially alter these properties, leading to protein inactivation, misfolding, or disrupted protein-protein interactions [7, 8].

Computational prediction of mutational effects has emerged as a powerful approach to guide experimental efforts and accelerate protein engineering campaigns. Traditional physics-based methods, such as Rosetta [9] and FoldX [10] employ energy functions to estimate stability changes but are often limited by computational speed and parametrization accuracy. Recent advances in machine learning, particularly deep learning, have shown promising results in predicting mutation effects [11–13]. However, most existing methods are either computationally expensive, require substantial expertise to operate, or focus on a single prediction task. Most importantly, many newly developed machine learning models lack validation through real-world protein engineering campaigns, leaving their practical utility uncertain.

To address these challenges, we recently developed Pythia, a self-supervised graph neural network for predicting mutation-induced changes in protein folding stability [14], and Pythia-PPI, an extension for predicting how mutations alter protein-protein binding affinity [16]. Pythia achieves state-of-the-art accuracy with computational speeds up to 10^5^-fold faster than conventional approaches, processing over 50 000 mutations per minute. Pythia-PPI combines transfer learning, multitask learning, and self-distillation strategies to achieve superior accuracy on protein-protein interaction datasets. These models have been validated through multiple protein engineering projects, demonstrating reliable performance in enzyme stabilization, evolution of activity, and antibody affinity maturation.

**Figure 1.**
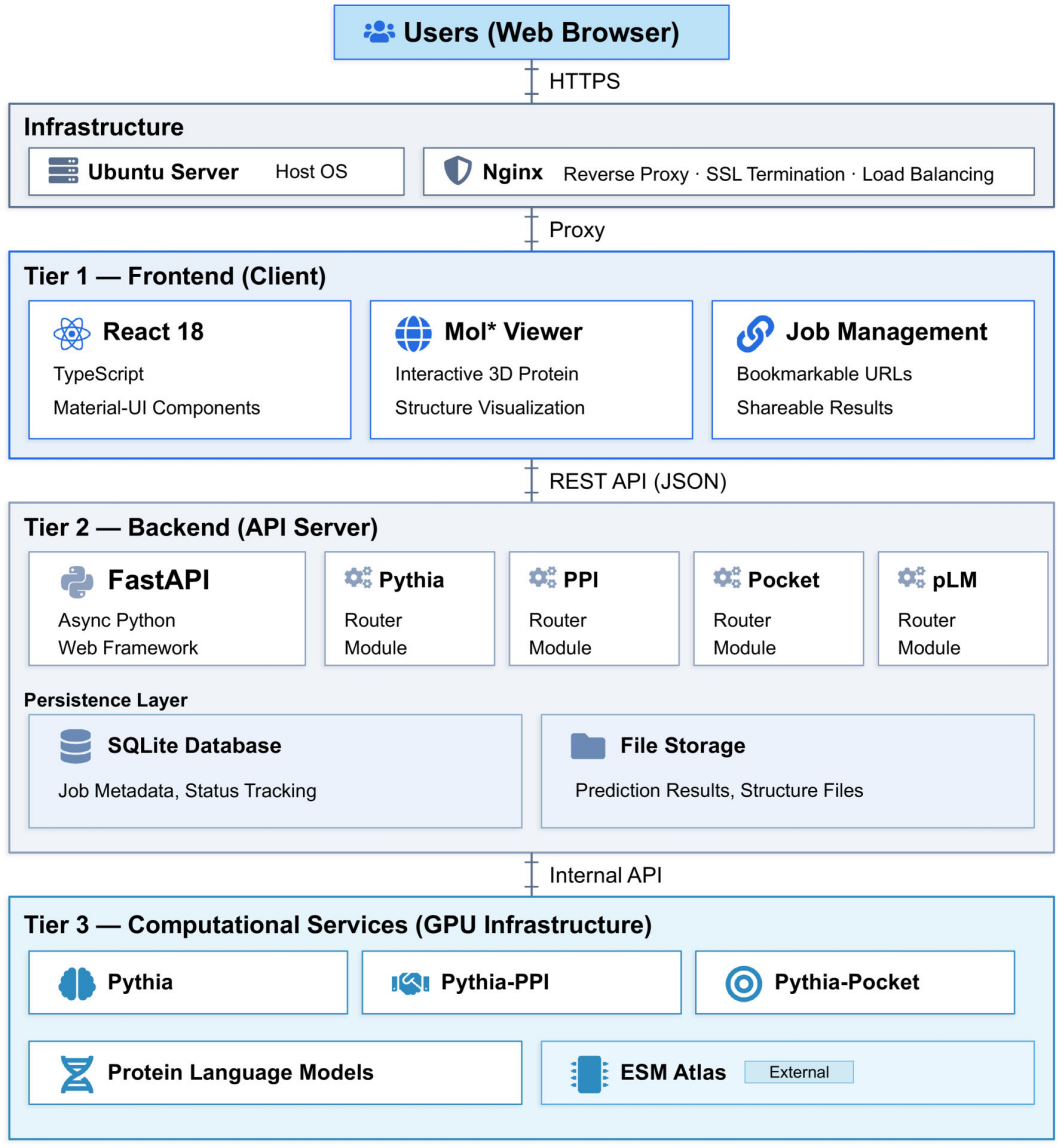
The architecture of the web server.

Encouraged by these real-world successes, we present PythiaStudio, a comprehensive web platform that makes these tools accessible to the broader scientific community. The platform adopts a three-tier architecture (Fig. 1) and integrates Pythia and Pythia-PPI with complementary analysis tools, including catalytic pocket identification via Pythia-Pocket, fitness prediction using protein language models, and structure prediction through ESMFold. Beyond individual predictions, PythiaStudio provides an engineering workflow that combines these tools for systematic protein optimization. We demonstrate the effectiveness of this integrated approach through a case study on glycoside hydrolase engineering, where the workflow successfully identified mutations that simultaneously improved thermostability and catalytic activity.

## Materials and methods

### Web server architecture

PythiaStudio is implemented as a three-tier web application (Fig. 1). The client tier is built with React 18 and TypeScript using Material-UI components, and incorporates the Mol* interactive 3D viewer for exploring structural features and mutation sites directly in the browser. The API server tier uses FastAPI with distinct router modules for Pythia, Pythia-PPI, Pythia-Pocket, and protein language model predictions, facilitating independent scaling. The computational services tier hosts GPU-accelerated prediction models internally, with ESMFold providing structure predictions as an external service. Job persistence relies on SQLite for metadata and file storage for prediction results; each completed job is assigned a unique identifier that produces a bookmarkable URL for revisiting or sharing. The entire stack is deployed on an Ubuntu server behind Nginx for SSL termination and load balancing.

### Underlying prediction models

**Figure 2.**
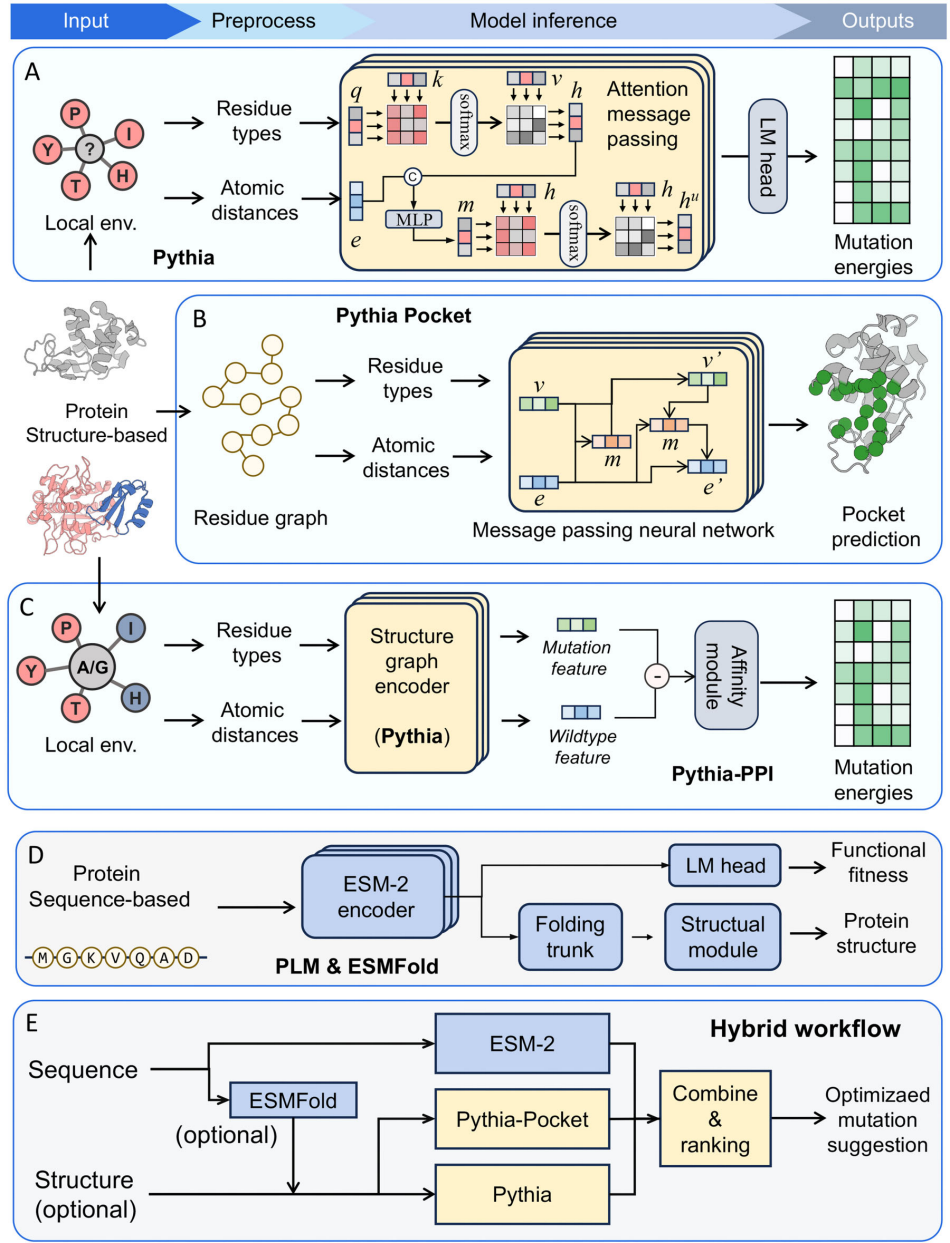
Overview of models and integrated workflow of the PythiaStudio web server. (**A**) Model architecture of Pythia. Residue and geometric features are processed through stacked attention-based message-passing layers, followed by a language-model-style head that estimates mutation energies from learned amino-acid probabilities. (**B**) Pythia-Pocket for functional residue identification. Protein structures are converted into residue graphs and analyzed using a message-passing neural network to identify residues likely involved in catalytic or binding pockets, enabling users to exclude functionally critical sites during design. (**C**) Pythia-PPI mutation effect prediction. Local protein structural environments are represented as residue graphs encoding residue types and atomic distances. These graphs are processed by the Pythia structure graph encoder to generate wild-type and mutant features, which are used to predict mutation-induced changes in protein–protein binding affinity (ΔΔG_bind_). Results are reported as comprehensive mutation energy matrices. (**D**) Sequence-based prediction modules. Protein language models are used for complementary analyses, including evolutionary fitness prediction with ESM-2 and structure prediction with ESMFold, which provides predicted structures when experimental structures are unavailable. (**E**) The inclusive engineering workflow of PythiaStudio. Sequence and/or structure inputs are flexibly routed through Pythia, Pythia-Pocket, ESM-2, and ESMFold. Predictions from multiple modules are combined and ranked to generate optimized mutation suggestions, enabling an end-to-end workflow from input to rational protein design.

PythiaStudio integrates five computational tools for protein analysis. Pythia is a self-supervised graph neural network trained on protein structures from the CATH database and RCSB PDB [14]. As illustrated in Fig. 2A, the model processes local protein structure as a k-nearest neighbor graph of Cα atoms and predicts amino acid probabilities at each position through stacked attention-based message-passing layers. The relationship between these probabilities and folding free energy changes follows from the Boltzmann distribution: ΔΔG ∝ $ - {\mathrm{ln}}( {{{{\mathrm{P}}}_{{\mathrm{mut}}}}/{{{\mathrm{P}}}_{{\mathrm{wt}}}}} )$. As Pythia is a self-supervised model that is not trained on ΔΔG mutation data, it avoids potential data leakage across benchmark datasets. Its performance on S669 is reported in Table 1 including Spearman’s correlation, ${{r}_{d - i}}$and $\langle \delta \rangle $. The ${{r}_{d - i}}$is defined as the Pearson correlation between predicted ΔΔG values for forward and reverse mutations:


\begin{eqnarray*}
{{r}_{d - i}} = {\mathrm{corr}}\left( {{\mathrm{\Delta \Delta }}{{G}_{{\mathrm{wt}}\to {\mathrm{mut}}}},{\mathrm{\ }}{\mathrm{\Delta \Delta }}{{G}_{{\mathrm{mut}}\to {\mathrm{wt}}}}} \right).
\end{eqnarray*}

For a physically consistent model, these values should satisfy:


\begin{eqnarray*}
{\mathrm{\Delta \Delta }}{{G}_{{\mathrm{wt}}\to {\mathrm{mut}}}} \approx - {\mathrm{\Delta \Delta }}{{G}_{{\mathrm{mut}}\to {\mathrm{wt}}}},
\end{eqnarray*}

and therefore ${{r}_{d - i}}$is expected to approach − 1.

$\langle \delta \rangle $ quantifies the average deviation from this ideal antisymmetry:


\begin{eqnarray*}
\langle \delta \rangle = \frac{1}{N}\mathop \sum \limits_{i = 1}^N \mid {\mathrm{\Delta \Delta }}G_{{\mathrm{wt}}\to {\mathrm{mut}}}^{\left( i \right)} + {\mathrm{\Delta \Delta }}G_{{\mathrm{mut}}\to {\mathrm{wt}}}^{\left( i \right)}\mid .
\end{eqnarray*}

**Table 1.** Comparison of Pythia with existing methods on the S669 dataset

	Spearman’s rho			Antisymmetry	
Method	Total	Direct	Inverse	r_d−i_	⟨δ⟩
**Pythia (fixed backbone**)	**0.66**	**0.46**	**0.46**	**−1**	**0**
Pythia (remodeled structure)	0.65	0.46	0.45	−0.84	1.01
PremPS [29]	0.63	0.42	0.42	−0.82	0.38
ACDC-NN [30]	0.63	0.45	0.44	−0.99	0.09
INPS3D [31]	0.57	0.44	0.36	−0.46	1.02
Dynamut [32]	0.48	0.37	0.36	−0.55	0.62
ThermoNet [13]	0.46	0.37	0.34	−0.83	0.31
PopMusic [33]	0.43	0.41	0.22	−0.23	1.42
DUET [34]	0.39	0.42	0.23	−0.07	1.48
MAESTRO [35]	0.39	0.46	0.19	−0.17	1.32
I-Mutant3.0 [36]	0.28	0.35	0.15	−0.01	1.62
DDGun [37]	0.59	0.43	0.41	−0.98	0.12
DDGun3D [37]	0.55	0.43	0.41	−0.97	0.18
mCSM [38]	0.34	0.36	0.21	−0.02	1.75
SDM [39]	0.28	0.39	0.14	−0.42	0.97

Spearman’s rho on the S669 dataset. Results taken from [14]. ${{r}_{d - i}}$: the Pearson correlation between predicted ΔΔG for forward (direct) and reverse (inverse) mutations. A value close to − 1 indicates ideal physical consistency (i.e. ΔΔG_wt→mut_ ≈ −ΔΔG_mut→wt_). ⟨δ⟩: a bias score measuring systematic deviation from perfect antisymmetry. Values close to 0 indicate low bias.

Lower values of $\langle \delta \rangle $ indicate smaller systematic bias and better adherence to thermodynamic constraints, with an ideal value of 0. Further evaluation on the S2648 and MegaScale datasets [15] has been previously reported [14]. Another unique feature of Pythia is the ultrafast speed; it is about 625–50 000–fold faster than conventional tools. See detailed speed benchmark in Supplementary Section 3, Figs S5–S6.

Pythia-PPI extends the Pythia architecture for protein–protein binding affinity prediction (Fig. 2C). The model employs transfer learning from the pretrained Pythia structure graph encoder, combined with multitask learning that simultaneously predicts binding affinity changes and protein stability changes. A self-distillation strategy expands the training data from approximately 4000 to 400 000 samples by generating predictions on all protein–protein interfaces in the SKEMPI v2 dataset [16], and the final model achieves a Pearson correlation of 0.79 (Table 2). The test is conducted as cross-validation, which is identical for all tested supervised models [17].

**Table 2.** Comparison of Pythia-PPI with existing methods

Method	Per-structure		Overall				
	Pearson	Spearman	Pearson	Spearman	RMSE	MAE	AUROC
Rosetta [9]	0.33	0.30	0.31	0.35	1.62	1.13	0.66
FoldX [10]	0.38	0.37	0.31	0.41	1.91	1.31	0.66
ESM-1v [12]	0.01	−0.01	0.19	0.16	1.96	1.37	0.54
ESM 3 [40]	0.08	0.07	0.21	0.14	4.59	1.52	0.54
MSA Transformer [41]	0.10	0.09	0.12	0.13	1.98	1.38	0.58
DDGPred [42]	0.38	0.34	0.66	0.47	1.50	1.08	0.70
RDE-Net [43]	0.44	0.40	0.64	0.56	1.58	1.11	0.75
Pythia-PPI [17]	0.57	0.53	0.79	0.64	1.08	0.74	0.78

Pythia-Pocket is a message-passing neural network trained on PDBBind to predict catalytic pocket residues (Fig. 2B). The precision is 0.408, recall is 0.462, AUROC is 0.809, and top-20 success rate is 0.766 when benchmarked on the LIGYSIS dataset [18], and achieved a balanced performance with both precision and recall at comparable levels. The detailed benchmark is in the Supplementary Section 4, Tables S1–S2, Fig. S7.

In addition to the core prediction modules, PythiaStudio also provides more tools to address broader protein engineering needs. ESM-2 provides evolutionary fitness predictions using masked language modeling on protein sequences [19, 20], while ESMFold generates structure predictions directly from amino acid sequences when experimental structures are unavailable (Fig. 2D). The indel scoring module uses the ProGen2-medium protein language model [21] to evaluate the fitness impact of single-residue deletions and alanine insertions. For each candidate sequence, the module computes the negative log-likelihood in both the N-to-C and C-to-N directions using the autoregressive model, averages the two values to obtain a bidirectional score, and reports the difference relative to the wild-type sequence as the indel fitness score. An aggregation prediction module, powered by Aggrescan3D 2.0 [22], enables users to analyze aggregation propensity and evaluate mutation effects on protein solubility.

### Input formats and processing

PythiaStudio accepts protein structures in PDB or mmCIF format for structure-based predictions and amino acid sequences in FASTA format for sequence-based predictions. It should be noted that the quality of input structures has a considerable impact on prediction reliability. For predicted structures, we recommend using high-confidence models generated by advanced structure prediction models such as AlphaFold [23]. We demonstrate how structure quality will affect the prediction accuracy in the Supplementary Section 1, Figs S1–S3. The platform automatically validates uploaded files and returns informative error messages when issues are detected. For users without pre-existing structural data, the integrated ESMFold module can generate predicted structures directly from sequences. For ESMFold, the length limit of the input sequence is 400. For ESM-2, the length limit is 2000. For indel prediction using ProGen2- medium, the length limit is 1022.

### Output visualization and export

All prediction tools in PythiaStudio provide comprehensive output visualization. Structure can be analyzed by the Mol* viewer [24]. Results are displayed as interactive heatmaps where users can visualize predicted effects across all possible single-point mutations at each position. Hovering over heatmap cells displays detailed mutation information and predicted values. Interactive sortable tables allow users to filter and search for specific mutations of interest. The top beneficial and detrimental mutations are automatically highlighted for quick identification. All results can be downloaded as CSV files for further analysis. In addition, users can select individual mutations of interest via checkboxes, hide unwanted entries to focus on the most promising candidates, and export either selected or all visible rows directly to Excel (.xlsx) format.

## Results

### Web server interface and workflow

PythiaStudio (https://pythiastudio.wulab.xyz/) is freely available to all users with no login requirement. The platform integrates five protein analysis tools through an intuitive interface (Fig. 1). The homepage provides direct access to Pythia for stability prediction, Pythia-PPI for binding affinity prediction, Pythia-Pocket for catalytic residue identification, ESM-2 for evolutionary fitness prediction, and ESMFold for structure prediction, each of which can be used independently or combined through the integrated engineering workflow.

The web interface accepts protein structures in PDB format, which can be downloaded directly from the RCSB Protein Data Bank. For proteins without experimental structures, users can upload structures predicted by AlphaFold2 [23] or similar tools, or use the integrated ESMFold module to generate predictions from amino acid sequences in FASTA format. When submitting Pythia, Pythia-PPI, or PLM fitness prediction tasks, the server performs comprehensive saturation mutagenesis screening across all possible single-point mutations. The low computational cost of the underlying models enables complete mutation landscapes to be returned within minutes, eliminating the need for users to repeatedly upload structures or manually define individual mutations.

For workflow tasks, users provide either a sequence or a structure. If only a sequence is provided, ESMFold first predicts the structure. PythiaStudio then runs Pythia, Pythia-Pocket, and ESM-2 to predict binding site residues, stability effects, and fitness scores. Results are integrated to provide a ranked list of mutations that balance stability and function, along with predicted pocket likelihoods. Each completed job receives a unique tracking URL for bookmarking and sharing with collaborators.

### Interactive result visualization

PythiaStudio presents prediction results through multiple complementary visualizations. The results of Pythia and Pythia-PPI primary display an interactive heatmap of 20 amino acid types by N protein positions, where color intensity indicates the magnitude of predicted values. For example, stabilizing mutations predicted by Pythia are shown in blue and destabilizing mutations in red. An accompanying sortable table supports filtering by position, mutation type, or predicted value, and automatically highlights the top stabilizing and destabilizing mutations. For Pythia-PPI, the webpage has a dedicated chain filter dropdown for the selection of a chain of interests. The integrated Mol* structure viewer allows users to examine mutation sites in a three-dimensional context. For Pythia-Pocket, the structures are colored by predicted pocket likelihood. All results can be downloaded as CSV files for downstream analysis.

### Complementary prediction tools

Pythia predicts protein stability change upon mutations, Pythia-PPI predicts protein binding affinity change upon mutations, and Pythia-Pocket predicts residues likely involved in catalytic function or ligand binding. When designing stability-enhancing mutations, users can exclude pocket residues identified by this tool to avoid compromising enzymatic activity or binding function. The tool displays predictions as colored residues overlaid on the structure viewer, with downloadable CSV files listing predicted pocket residues and confidence scores.

ESM-2 provides evolutionary fitness predictions in a zero-shot manner. Like Pythia, this tool returns log-likelihood ratio scores for all possible mutations, where more negative scores indicate mutations predicted to maintain or improve function based on evolutionary constraints. We recommend users to use the default ESM-2 (650M) model, as it ranks first among all zero-shot single-sequence models. For more details, refer to the Supplementary Section 2, Fig. S4.

ESMFold enables structure prediction from sequence when experimental structures are unavailable. Users submit protein sequences in FASTA format, and the server returns predicted structures. The predicted Local Distance Difference Test (pLDDT) score averaged on Cα atoms was shown to indicate the confidence of prediction. The predicted structures can be directly used as input for Pythia or Pythia-Pocket, enabling the complete prediction workflow to start from the sequence alone.

### Engineering workflow for protein optimization

Beyond individual prediction tools, PythiaStudio provides an integrated engineering workflow that systematically combines stability and fitness predictions, as illustrated in Fig. 2E. This workflow addresses a common challenge in protein engineering: mutations that improve one property often compromise another. The two-step approach first identifies thermostabilizing mutations using Pythia, then uses ESM-2 to discover activity-enhancing mutations in the stabilized background.

The workflow begins with structure input, either uploaded by the user or generated via ESMFold. Pythia generates a comprehensive stability landscape, and users select candidate stabilizing mutations with predicted ΔΔG values that fall below −3.0. These candidates are experimentally validated, and beneficial mutations are accumulated using a greedy strategy. Once a stabilized variant is obtained, ESM-2 identifies mutations with fitness scores below −1.5 that are predicted to enhance function. These thresholds were empirically chosen based on our previous experimental engineering campaigns [25] and should be interpreted as recommended starting points rather than universal cutoffs; users are encouraged to adjust them according to their specific experimental throughput and validation capacity. This sequential approach mitigates stability–activity trade-offs that frequently limit protein engineering success.

### Case study: glycoside hydrolase engineering

We previously validated the PythiaStudio engineering workflow by applying it to *Bacillus subtilis* cellulase and β-glucanase [25], two industrially relevant glycoside hydrolases whose application in biomass processing is limited by insufficient thermostability. Engineering these enzymes is challenging because stabilizing mutations often reduce catalytic activity by rigidifying regions required for substrate binding.

In the first step, Pythia screened all possible single-point mutations with predicted catalytic and substrate-binding residues excluded. Mutations with Pythia scores below −3.0 were prioritized. For cellulase, greedy accumulation of three stabilizing mutations yielded cellulase-M2 (ΔTm = 3.9°C, 32% increased activity). For β-glucanase, five mutations were accumulated to generate β-glucanase-M5 (ΔTm = 7.3°C, wild-type activity retained). In the second step, ESM-2 fitness predictions identified activity-enhancing mutations in the stabilized backgrounds: cellulase-M5 achieved ΔTm = 5.8°C with 1.5-fold activity, and β-glucanase-M7 achieved ΔTm = 8.4°C with 1.1-fold activity improvement.

Structural analysis revealed complementary stabilization mechanisms: Pythia-identified mutations stabilized peripheral scaffold regions, whereas ESM-2-identified mutations optimized substrate-binding regions. The final variants showed substantially improved performance, with cellulase-M5 retaining full activity at 50°C over 24 h with a 2.2-fold higher *k*_cat_, while β-glucanase-M7 achieved ΔTm = 8.4°C with a 2.2-fold extended half-life and improved substrate affinity, demonstrating that the two-step workflow effectively overcomes the stability–activity trade-off in enzyme engineering. A similar workflow has also been applied to additional enzymes, including amidase [26], galactosidase [27], and transaminase [28], further validating its generality.

## Discussion

PythiaStudio provides an integrated protein engineering platform centered on the Pythia model family, including Pythia for stability prediction, Pythia-PPI for binding affinity prediction, and Pythia-Pocket for pocket residue identification, complemented by additional modules for fitness, indel, aggregation, and structure prediction. The efficiency of the underlying models enables rapid saturation mutagenesis screening within seconds, making them substantially faster than traditional physics-based approaches. The unified enzyme engineering workflow enables end-to-end mutation design within a single platform, allowing users to jointly consider stability and activity-related properties. This is illustrated by the glycoside hydrolase case study, where combining Pythia and ESM-2 improved both thermostability and catalytic activity.

Prediction accuracy depends on the quality of input structures, and users should employ high-confidence models when using AlphaFold2 or ESMFold predictions. Future developments will include batch processing for protein families, multi-mutation analysis, and specialized workflows for antibody optimization. We anticipate that PythiaStudio will serve as a useful and complementary platform for protein engineering in both academic and industrial settings.

## Supplementary Material

gkag408_Supplemental_File

## Data Availability

PythiaStudio is freely accessible at https://pythiastudio.wulab.xyz/. The source code for the underlying Pythia and Pythia-Pocket models is available at https://github.com/Wublab/pythia (https://doi.org/10.5281/zenodo.19589355), and Pythia-PPI source code is available at https://github.com/Wublab/pythia_ppi (https://doi.org/10.5281/zenodo.19589367). The web server does not require registration for use.
