# Supplementary material for: PythiaStudio: a one-stop protein engineering platform powered by Pythia model suite
Source: Nucleic Acids Res. 2026 May 15;54(W1):W306–13. doi: 10.1093/nar/gkag408 (PMC13355049; doi:10.1093/nar/gkag408)
Supplement: gkag408_Supplemental_File [file gkag408_supplemental_file.docx]

**Supporting information:**

**PythiaStudio: a one-stop protein engineering platform powered by Pythia model suite**

Jinyuan Sun^1,2,†^, Kelun Shi^1,2,†^, Han Li^5,†^, Yinglu Cui^1,4,*^, Luoyi Wang^1,4,*^ and Bian Wu^1,2,3,*^

1 *State Key Laboratory of Microbial Diversity and Innovative Utilization, Institute of Microbiology, Chinese Academy of Sciences, Beijing 100101, China*

2 *College of Life Sciences, University of Chinese Academy of Sciences, Beijing 100049, China*

3 *State Key Laboratory of Green Biomanufacturing, Beijing University of Chemical Technology, Beijing 100029, China*

4 *Beijing Key Laboratory of Genetic Element Biosourcing & Intelligent Design for Biomanufacturing, Beijing, China*

5 *Independent Researcher*

* To whom correspondence should be addressed. Email: thebianwu@outlook.com. Correspondence may also be addressed to wangluoyi@im.ac.cn (Luoyi Wang) and cuiyinglu@im.ac.cn (Yinglu Cui)

† These authors contributed equally to this work.

**1. Structural Quality**

Since PythiaStudio relies on protein structures as input for its core prediction modules, the quality of input structures can considerably affect prediction reliability. We conducted a systematic analysis of this effect using stability datasets, and here we summarize the key findings to guide users of PythiaStudio.

On the X-ray solved subset of S2648 (2,306 mutations), predictions using AlphaFold2- or ESMFold-predicted structures matched or even slightly surpassed those using experimental PDB structures (Spearman's ρ = 0.612, 0.603, and 0.588 for AlphaFold2, ESMFold, and PDB, respectively), consistent with earlier observations by Akdel et al. (1). However, analysis on the larger mega-scale dataset revealed that prediction accuracy is positively correlated with model confidence scores (pLDDT) for both AlphaFold2 and ESMFold (Supplementary Figure S1). Structures with higher pLDDT values tend to yield higher per-protein Spearman's ρ, while low-confidence predictions can substantially degrade performance.


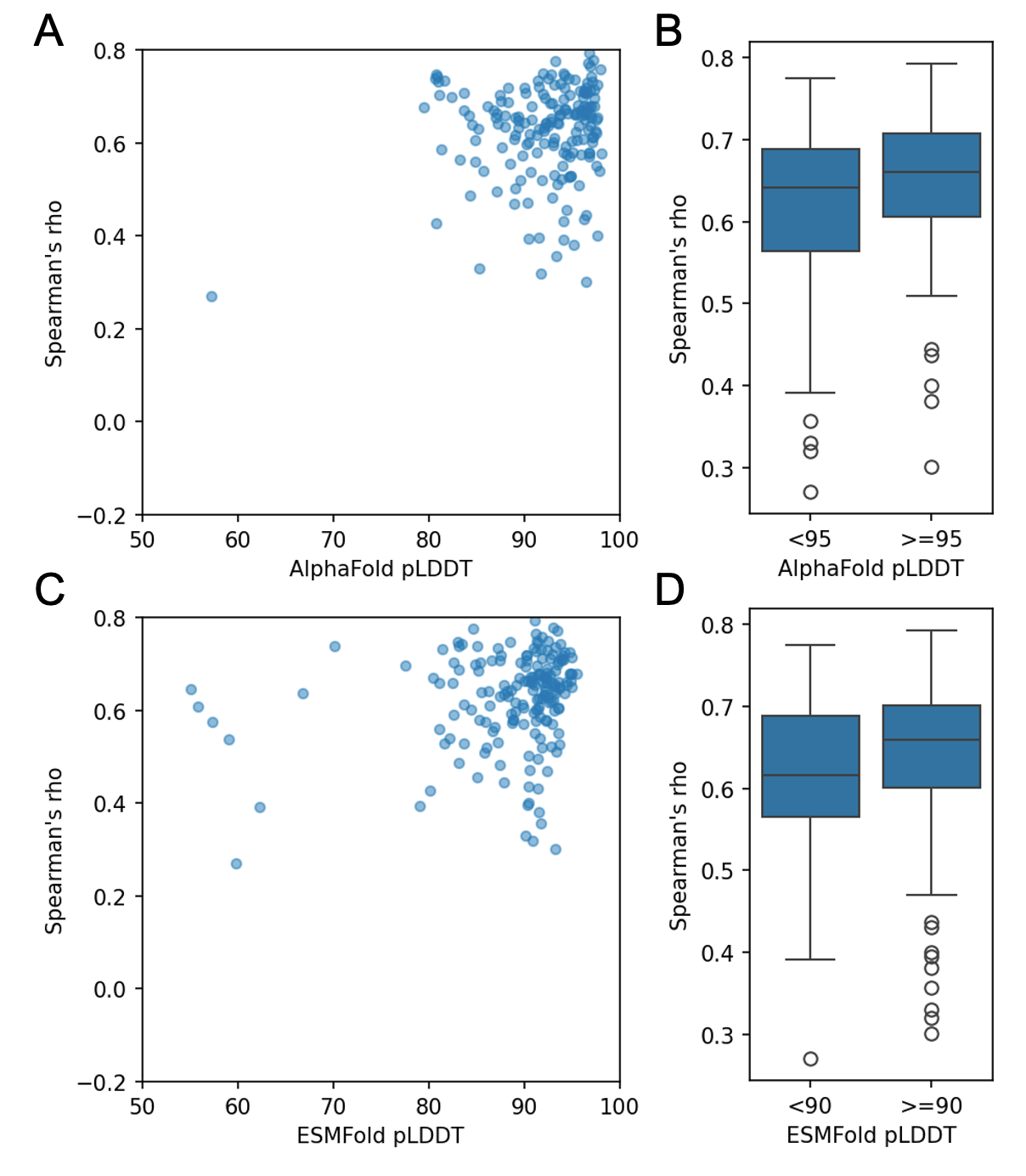


Supplementary Figure S1. Relationship between prediction accuracy and pLDDT of predicted structures. (A) Scatter plot of per-protein Spearman's ρ versus mean pLDDT for AlphaFold2-predicted structures. (B) Boxplot comparison of Spearman's ρ for AlphaFold2 structures with pLDDT < 95 versus ≥ 95. (C) Scatter plot of per-protein Spearman's ρ versus mean pLDDT for ESMFold-predicted structures. (D) Boxplot comparison of Spearman's ρ for ESMFold structures with pLDDT < 90 versus ≥ 90.

Two representative failure modes illustrate this effect. In the first case (PDB ID: 2MC5), AlphaFold2 produced a structure with a low mean pLDDT of 57 and an RMSD of 5.99 Å to the experimental X-ray structure, resulting in a poor Spearman's ρ of 0.270; switching to the X-ray structure improved the correlation to 0.484 (Supplementary Figure S2).


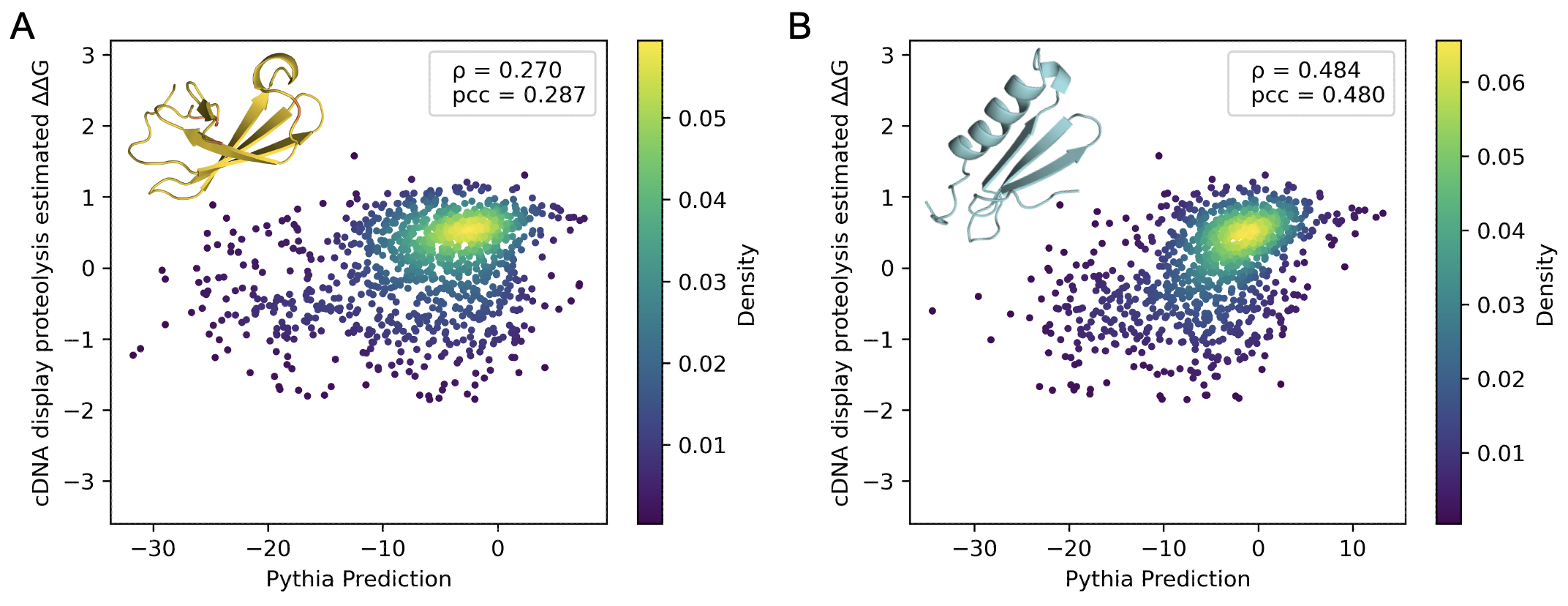


Supplementary Figure S2. Density scatter plots of Pythia predictions versus cDNA display proteolysis-estimated ΔΔG for protein 2MC5 using different structural inputs. (A) Prediction using the AlphaFold2-predicted structure, shown as cartoon at the upper left and colored by pLDDT (yellow indicates scores of 50–70). The AlphaFold2 model has a mean pLDDT of 57 and an RMSD of 5.99 Å to the X-ray structure, yielding a Spearman's ρ of 0.270. (B) Prediction using the experimentally determined X-ray structure, shown as cartoon colored cyan, yielding an improved Spearman's ρ of 0.484. Note that the ESMFold-predicted structure for this protein was also inaccurate.

In the second case (PDB ID: 6OBK), AlphaFold2 generated a model with high pLDDT but mispositioned an alpha-helix relative to the NMR structure, yielding a Spearman's ρ of only 0.302. In this instance, the ESMFold-predicted structure was closer to the experimentally determined fold, and using it as input improved the correlation to 0.687 (Supplementary Figure S3).

Supplementary Figure S3. Density scatter plots of Pythia predictions versus cDNA display proteolysis-estimated ΔΔG for protein 6OBK using different structural models. (A) Prediction using the AlphaFold2-predicted structure (green), superimposed with the NMR structure (magenta). The mispositioned alpha-helix in the AlphaFold2 model leads to a Spearman's ρ of 0.302. (B) Prediction using the ESMFold-predicted structure (cyan), superimposed with the NMR structure (magenta), yielding an improved Spearman's ρ of 0.687.

These results highlight that no single structure prediction method is universally superior. We recommend the following guidelines for PythiaStudio users: (i) whenever available, use experimentally determined structures from the PDB; (ii) when experimental structures are unavailable, use high-confidence predicted structures from AlphaFold2/3 or comparable tools such as Protenix, and inspect the pLDDT scores to ensure overall model confidence is high (e.g., mean pLDDT ≥ 90); (iii) for critical targets, consider comparing predictions obtained from multiple structural sources to assess robustness; and (iv) exercise particular caution with proteins that lack close homologs in training databases, as structure prediction tools may be less reliable for these cases.

**2. ESM-2 Fitness Prediction**

PythiaStudio integrates ESM-2, a series of masked protein language models (2), to enable zero-shot fitness prediction for all possible single-point substitutions. Below, we describe the scoring methodology, interpretation, and benchmark performance.

We adopt the masked marginal scoring strategy, which was shown to outperform alternative scoring schemes including wild-type marginals and pseudolikelihood. For each position i in the input sequence, the residue is replaced with a [MASK] token and the model outputs a probability distribution over all 20 standard amino acids via a softmax layer.

The fitness score for a substitution from wild-type residue x_wt_ to mutant residue x_mut_ at position i is computed as the negative log-likelihood ratio:

$$\text{score}=- (\log P(x_{\mathrm{mut}}\mid x_{i})-\log P(x_{\mathrm{wt}}\mid x_{i})),$$

where **x**_i_ denotes the sequence context with position i masked.

Because the probabilities are softmax-normalized, the score reflects how the ESM model, which trained on millions of evolutionarily diverse sequences, evaluates the relative plausibility of mutant versus wild-type residues in a given sequence context. A negative score indicates that the mutant residue is more likely than the wild type, whereas a positive score suggests a less favorable substitution. Scores close to zero indicate approximately neutral effects. In practice, scores typically fall within a range of approximately −5 to +15, although more extreme values can occur at highly conserved or highly variable positions.

ESM-2 has been extensively evaluated on the ProteinGym benchmark (3), which includes 217 deep mutational scanning assays covering ~2.7 million variants across diverse proteins and functional readouts including activity, binding, expression, organismal fitness, and stability. On the single-substitution benchmark, ESM-2 (650M) achieves a mean Spearman’s correlation of **0.422** using the masked marginal strategy, ranking first among all zero-shot single-sequence models and outperforming larger ESM-2 variants as well as other model families (Figure S4).

These results highlight several practical advantages of ESM-2 for web-based applications. Firstly, it requires only a single input sequence. Second, inference is efficient, enabling full saturation mutagenesis scans within minutes. Thrid, its predictions have been validated in experimental protein engineering studies for identifying activity-enhancing mutations.


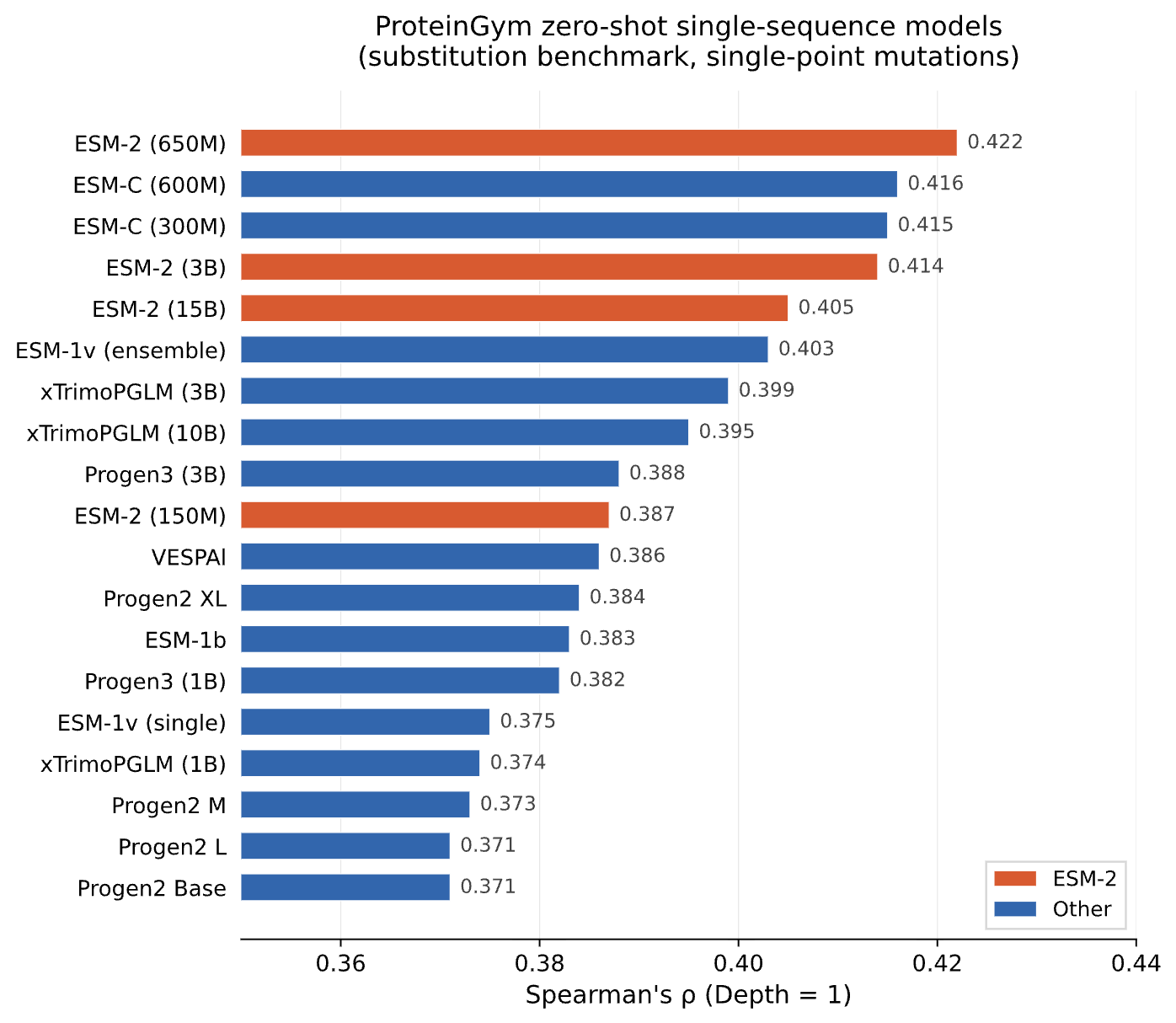


Supplementary Figure S4. Spearman's ρ of zero-shot single-sequence models on the ProteinGym signle substitution benchmark. ESM-2 variants are shown in red. ESM-2 (650M) ranks first (ρ = 0.422), outperforming larger ESM-2 checkpoints and all other model families. Data from ProteinGym v1.3.

**3. Speed test of Pythia model**

We conducted a benchmark analysis of computational efficiency for FoldX (5) and Pythia, assessing their runtimes for 385,776 mutations across 111 proteins with lengths ranging from 40 to 554 residues (Figure S5). When Pythia is executed sequentially, without prior batching of structures, it requires 570 CPU seconds to complete the computations. In contrast, FoldX demands a significantly longer 21,703,680 CPU seconds, demonstrating that Pythia is approximately 38,000 times faster than FoldX. Furthermore, when the efficiency of Pythia could be enhanced through prior batching of structures before being passed into the neural network, the required computational time is reduced to 210 CPU seconds, achieving approximately 10^5^-fold speedup.


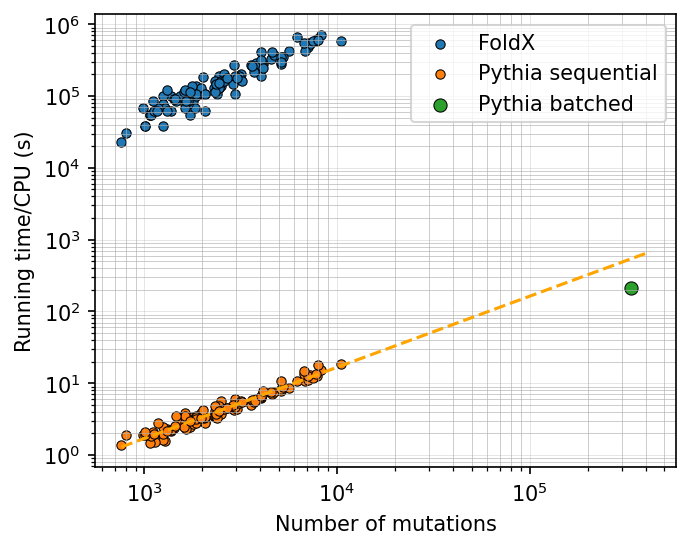


**Supplementary Figure S5.** Comparison of computational efficiency between Pythia and FoldX for saturation mutagenesis. The scatter plot shows runtime as a function of protein length. FoldX (blue) exhibits runtimes that scale steeply with protein length, whereas Pythia in sequential mode (orange) scales much more slowly. The dashed orange line indicates the extrapolated trend for Pythia sequential execution. Pythia with batched structure preprocessing (green) further reduces runtime, achieving an overall speedup of approximately 10⁵-fold compared to FoldX.

Additionally, we further compared the speed of other methods based on neural networks that can be accelerated by GPU. Pythia is about 62 times faster than MIF (5) and 3,000 times faster than ESM-IF (6) (Figure S6). By employing fewer local feature representations, the computational resource requirement is further optimized due to less preprocessing computation. Consequently, the computational demand for a single mutation within the neural network remains consistent regardless of an increase in protein length.


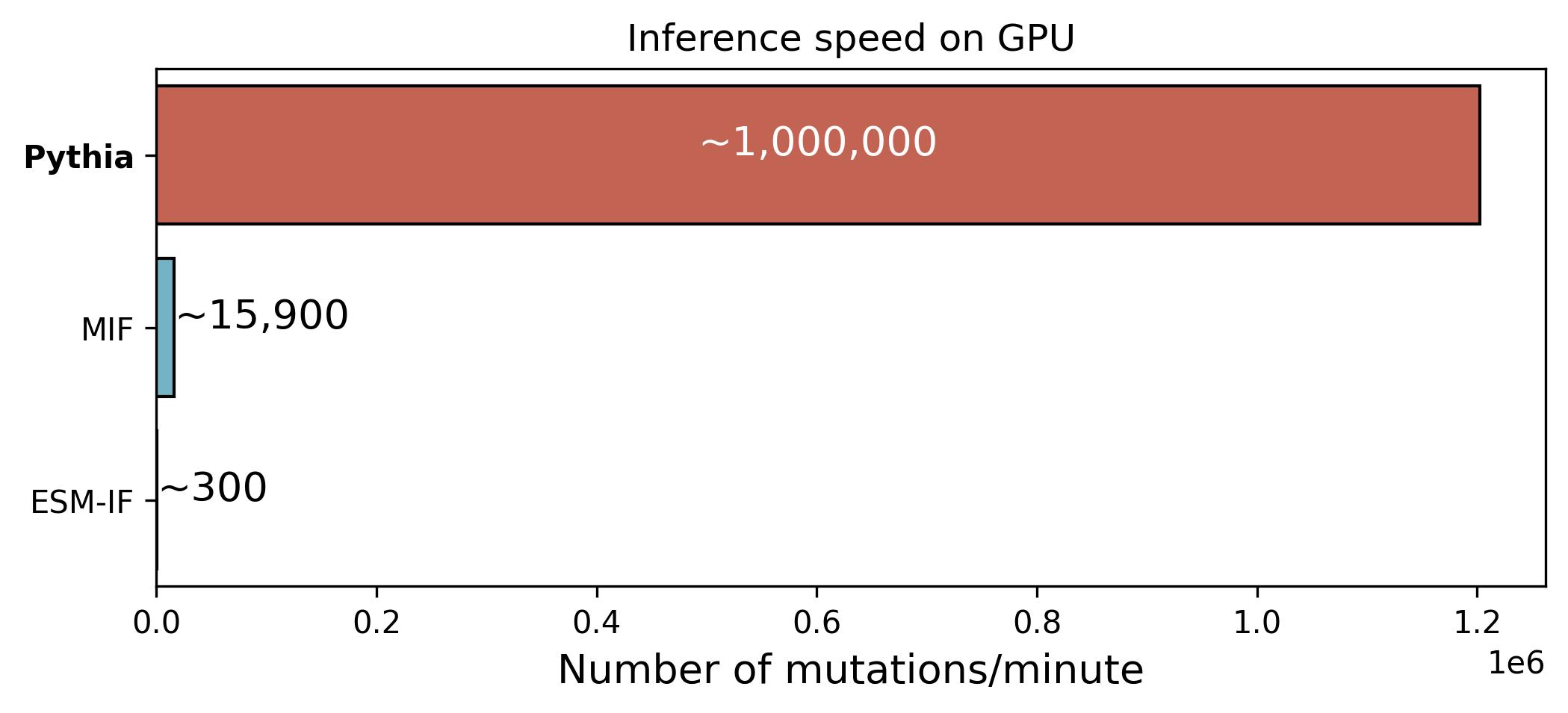


**Supplementary Figure S6.** Comparison of GPU inference speed among structure-based neural network methods. Pythia achieves approximately 1,000,000 mutations per minute, compared to ~15,900 for MIF and ~300 for ESM-IF, corresponding to ~62-fold and ~3,000-fold speedups, respectively. The substantially higher throughput of Pythia results from its use of compact local structural features, which minimizes preprocessing overhead and maintains constant per-mutation computational cost regardless of protein length.

**4. Benchmark of Pythia-Pocket**

To evaluate the performance of Pythia-Pocket, we benchmarked it on the LIGYSIS dataset (7), a curated collection of 2,367 representative protein chains with residue-level binding site annotations derived from experimentally observed protein–ligand interactions. We compared Pythia-Pocket against several established pocket prediction methods, including P2Rank (9), IF-SitePred (8), VN-EGNN (10), and fpocket (11).

We further developed Pythia-Pocket v1.1, which incorporates reduced sequence redundancy between training and evaluation data using MMseqs2 clustering at ninety percent sequence identity and eighty percent coverage. Chains without annotated pocket residues were excluded from training.

All methods were evaluated at the residue level. A residue was considered a positive pocket residue if it was annotated as part of a ligand-binding site in the LIGYSIS ground truth. We report both classification-based and ranking-based metrics.

For classification, we report precision, recall, F1-score, and Matthews correlation coefficient. Precision is defined as the fraction of predicted pocket residues that are true pocket residues,

$$\mathrm{Precision}=\frac{\mathrm{TP}}{\mathrm{TP}+\mathrm{FP}},$$

and recall measures the fraction of true pocket residues that are correctly identified,

$$\mathrm{Recall}=\frac{\mathrm{TP}}{\mathrm{TP}+\mathrm{FN}}.$$

The F1-score is the harmonic mean of precision and recall,

$$F1=\frac{2\cdot\mathrm{Precision}\cdot\mathrm{Recall}}{\mathrm{Precision}+\mathrm{Recall}},$$

and the Matthews correlation coefficient provides a balanced measure that accounts for all four entries of the confusion matrix,

$$\mathrm{MCC}=\frac{\mathrm{TP}\cdot\mathrm{TN}-\mathrm{FP}\cdot\mathrm{FN}}{\sqrt{\left( \mathrm{TP}+\mathrm{FP})(\mathrm{TP}+\mathrm{FN})(\mathrm{TN}+\mathrm{FP})(\mathrm{TN}+\mathrm{FN} \right)}}.$$

We first report pooled metrics across all residues in Table S1. Under this setting, Pythia-Pocket v1.1 achieves the highest F1-score and MCC among the compared methods, indicating the best overall classification performance. In contrast, fpocket achieves high recall but substantially lower precision, whereas VN-EGNN shows the opposite trend, reflecting different operating regimes.

| Table S1 Pooled metrics on the LIGYSIS dataset | | | | |
| --- | --- | --- | --- | --- |
| Method | F1 | MCC | Precision | Recall |
| Pythia-Pocket v1.1 | **0.433** | **0.370** | 0.408 | 0.462 |
| IF-SitePred | 0.412 | 0.345 | 0.339 | 0.525 |
| Pythia-Pocket v1 | 0.399 | 0.335 | 0.304 | **0.580** |
| P2Rank | 0.354 | 0.278 | 0.281 | 0.478 |
| VN-EGNN | 0.292 | 0.256 | **0.419** | 0.224 |
| fpocket | 0.219 | 0.120 | 0.130 | 0.695 |

To account for variability across protein chains, we further report macro-averaged metrics computed per chain in Table S2. Under this evaluation, performance differences between methods are reduced, although Pythia-Pocket models remain competitive across all metrics.

| Table S2 Averaged metrics on the LIGYSIS dataset | | | | |
| --- | --- | --- | --- | --- |
| Method | F1 | MCC | Precision | Recall |
| Pythia-Pocket v1 | **0.367 ± 0.224** | 0.280 ± 0.270 | 0.300 ± 0.211 | 0.605 ± 0.335 |
| Pythia-Pocket v1.1 | 0.334 ± 0.274 | **0.282 ± 0.294** | 0.316 ± 0.273 | 0.451 ± 0.384 |
| P2Rank | 0.325 ± 0.263 | 0.277 ± 0.271 | 0.321 ± 0.296 | 0.426 ± 0.323 |
| VN-EGNN | 0.304 ± 0.226 | 0.276 ± 0.272 | **0.516 ± 0.397** | 0.258 ± 0.216 |
| IF-SitePred | 0.301 ± 0.262 | 0.253 ± 0.265 | 0.250 ± 0.236 | 0.473 ± 0.402 |
| fpocket | 0.234 ± 0.154 | 0.120 ± 0.167 | 0.161 ± 0.130 | **0.645 ± 0.279** |

For ranking-based evaluation, we report receiver operating characteristic area under the curve (ROC-AUC) and precision–recall area under the curve (PR-AUC). The ROC curve is shown in Figure S7A and summarizes the trade-off between true positive rate and false positive rate across decision thresholds. The precision–recall curve is shown in Figure S7B and reflects performance under class imbalance. In both evaluations, Pythia-Pocket v1.1 achieves the strongest overall ranking performance. We also evaluated the Top-N success rate, defined as the fraction of protein chains for which at least one true pocket residue appears among the top-N ranked residues. The results are shown in Figure S7C. Pythia-Pocket v1.1 shows strong enrichment of true pocket residues, with high success rates even at small N. The precision–recall trade-off across methods is shown in Figure S7D. Different methods occupy distinct regions of the precision–recall space. Pythia-Pocket v1.1 achieves a balanced performance with both precision and recall at comparable levels.

**Supplementary Figure S7**. Benchmarking of pocket prediction methods on the LIGYSIS dataset (A) ROC curves for residue-level pocket prediction. Numbers in parentheses indicate ROC-AUC values. (B) PR curves for residue-level pocket prediction. Numbers in parentheses indicate PR-AUC. The dashed line indicates the random baseline. (C) Top-N success rate, defined as the fraction of protein chains for which at least one true pocket residue appears among the top N ranked residues. (D) Precision–recall trade-off across methods, showing precision and recall values at the selected decision threshold.

**Reference**

1. Akdel, M., Pires, D., Pardo, E. et al. (2022) A structural biology community assessment of AlphaFold2 applications. Nat. Struct. Mol. Biol., 29, 1056–1067. doi:10.1038/s41594-022-00849-w.
2. Lin, Z., Akin H., Rao R. et al. (2023) Evolutionary-scale prediction of atomic-level protein structure with a language model. Science, 379, 1123-1130. doi:10.1126/science.ade2574.
3. Notin, P., Kollasch A., Ritter D. et al. (2023) Proteingym: large-scale benchmarks for protein fitness prediction and design. BioRxiv, 12, 07, 570727. doi:10.1101/2023.12.07.570727.
4. Schymkowitz, J., Borg, J., Stricher, F. et al. (2005) The FoldX web server: an online force field. Nucleic Acids Res., 33, W382-W388. doi:10.1093/nar/gki387.
5. Yang, K., Niccolò Z., Hugh Yeh. (2023) Masked inverse folding with sequence transfer for protein representation learning. BioRxiv, 2022, 05, 25, 493516. doi:10.1101/2022.05.25.493516.
6. Hsu C., Verkuil R., Liu J. et al (2022) Learning inverse folding from millions of predicted structures. BioRxiv, 2022, 04, 10, 487779. doi:10.1101/2022.04.10.487779.
7. Utgés, J., Geoffrey J. (2024) . Comparative evaluation of methods for the prediction of protein–ligand binding sites. Journal of Cheminformatics, 16, 126. doi:10.1186/s13321-024-00923-z.
8. Carbery A., Buttenschoen M., Skyner R. et al (2024) Learnt representations of proteins can be used for accurate prediction of small molecule binding sites on experimentally determined and predicted protein structures. Journal of Cheminformatics, 16, 32. doi:10.1186/s13321-024-00821-4.

Krivák R., Hoksza D. (2018) P2Rank: machine learning based tool for rapid and accurate prediction of ligand binding sites from protein structure. Journal of Cheminformatics, 10, 39. doi:10.1186/s13321-018-0285-8.

Sestak, F., Schneckenreiter L., Brandstetter J. et al. (2025) VN-EGNN: E (3)-and SE (3)-Equivariant Graph Neural Networks with Virtual Nodes Enhance Protein Binding Site Identification. ArXiv, 2404, 07194. doi:arxiv-2404.07194

Guilloux V., Schmidtke P., Tuffery P. (2009) Fpocket: an open source platform for ligand pocket detection. BMC Bioinformatics, 10, 168. doi:10.1186/1471-2105-10-168.
